# Small pond, big opportunities: rethinking social mobility through niche sports in Latin America

**DOI:** 10.3389/fspor.2025.1684657

**Published:** 2025-12-05

**Authors:** Oliver Chen

**Affiliations:** Upper Canada College, Toronto, ON, Canada

**Keywords:** Latin America, sport-for-development, inequality, youth development, Bourdieu, football (soccer), badminton, social mobility

## Abstract

This study investigates whether niche sports, relative to mainstream ones, can better improve social mobility for marginalized youth in contemporary Latin America. Sports in general, especially mainstream sports, have often been presented as engines of empowerment for youth. However, critical analyses have shown that contrary to expectations, athletic pathways are often overcrowded, unevenly structured, and incapable of delivering broad mobility. Testing soccer as mainstream against badminton, this comparative study analyzes a core data-corpus of 47 media sources along with three semi-structured primary interviews; Bourdieu's field-capital framework and Sen's human capability approach lens guide the discussion. The study's results identify how smaller-scale sports hold mechanisms that effectively widen one's options. Specifically, badminton is found to (i) offer quicker visibility in a less congested field; (ii) improve simpler access to stipends, scholarships, and institutional posts; and (iii) increase gains in identity, confidence, and networks that translate into further opportunities. While badminton's economic upside is thinner relative to soccer, opportunities are more reachable and less dependent on prior advantage, a key aspect to achieving higher mobility. Overall, this study concludes that while badminton offers smaller absolute rewards than football, these rewards are more realistically obtainable due to greater accessibility and hold more long-term value for advancing social mobility. Thus far, this has been an overlooked route for challenging entrenched inequalities. As global sport becomes increasingly commercialized, this paper sets a foundation for expanding research on how niche sporting environments can offer more inclusive and effective pathways for social mobility in the Global South.

## 1 Introduction

Throughout Latin America, one of the world's most unequal regions, sport has become mobilized as a tool to promote youth development, social cohesion, and, in some cases, enable upward mobility. Evidence from global reviews suggests that sport can effectively contribute to developing factors like education, psychosocial well-being, and inclusion when embedded in sound pedagogy and supportive structures. Such effects, however, are often uneven and overly context-dependent (1). Specifically, critical scholarships in recent decades have cautioned that sport-for-development (SFD) initiatives often promise “broad-gauge” change, but limit through “limited-focus” interventions. This risks overstatement of impacts unless programmes are integrated with education, health, and social policy systems (2, 3). This paper contributes to the aforementioned debate in the Latin American context, where SFD has grown rapidly yet remains theoretically fragmented and unevenly distributed across sports and countries.

A global mapping of SFD organisations, including all international regions, finds football to be the most common delivery sport across multiple outcome domains. This is a recurring pattern that is mirrored in Latin America and frequently reproduced in scholarly attention (4, 5). Such concentration is crucial to be brought to attention, as large, highly competitive fields may heighten capital thresholds and selection pressures. This holds, as critical analyses have argued, the potential to lower the probability that most participants convert sport involvement into tangible social or educational gains. At the same time, it is important to note that football is not the only sport; Latin America's SFD ecosystem is evidently heterogeneous, with multilateral and national actors having invested across over 18 countries in the region. This spans from community-based programmes to facility development and athlete support for a wide range of sports (6). A notable example of this is Mexico's Comisión Nacional de Cultura Física y Deporte (CONADE), which administers athletic scholarship schemes. Furthermore, Paraguay's Secretaría Nacional de Deportes operates an elite multi-sport support programme, whereas Peru's Legado/VIDENA high-performance infrastructure supports a broad set of both Olympic and non-traditional sports (7–9). These diverse policy settings, paired alongside community initiatives like Rio's “Miratus” badminton project for underprivileged youth, illustrate that mainstream and niche sports and athlete systems coexist in Latin America (10). Crucially, with such diversity, they may present distinct opportunity structures for youth.

Against this backdrop, this study advances a theory-driven comparative analysis of how varying sporting fields may shape young people's real opportunities in different ways. Conceptually, Bourdieu's theory of fields and capitals is integrated with the analysis, with specific attention to the economic, cultural, social, and symbolic capital gained from sports as well as the congestion of each sport system's “field”. This is additionally paired alongside Sen's Capability Approach, a lens which evaluates development in terms of the substantive freedoms people have to achieve valued beings and doings (11, 12). These frameworks are applied through a methodological contrast of two cases, using football (mainstream) and badminton (niche). Such analysis enables a deeper investigation of how field size, pathways, and capital requirements condition the conversion of sport participation into capabilities. This study investigates a broadened regional base to develop claims for niche sports in Latin America as a whole, drawing on primary and literary sources from various regional countries. Specifically, personal narratives and quantitative indicators are explored from Mexico, Paraguay, Peru, Brazil, Guatemala, Argentina, and beyond. Inference limitations are acknowledged when evidence is thin or missing, and distinctions are explicitly made between narratives and empirical findings. Ultimately, this paper aims to open pathways for further research on niche sports in Latin America SFD, seeking to move beyond simple celebratory or dismissive accounts of sport's role seen in the general debate. To do this, I explore the question of whether and how niche sports, relative to mainstream ones, can generate more accessible forms of social mobility for marginalized youth in Latin America.

## 2 Literature review

In recent decades, scholars have deeply examined the correlation between sport participation and advanced social mobility for Latin American youth. Current literature nonetheless remains divided, with an emerging debate discussing the true extent of sport's effectiveness. Notable studies like (13) and (14) trace gains in identity, agency, and social ties; however, critics like (15) argue that persistent structural inequalities conversely blunt sport's influence on one's life trajectories. This paper aims to avoid a purely general overview by engaging this scholarship more directly to find a notable gap. Specifically, the literature review section is organized into three strands: (i) a synthesis of evidence showing identity gains and structural constraints as framed in current Latin American SFD literature for context [analyzing through the Positive Youth Development (PYD) framework and Bourdieusian power/field analyses that have thus far been utilized in studies]; (ii) an exploration into the predominance of mainstream sports (i.e., football) throughout programs and research and more importantly, why (if apparent) this imbalance may create limitations; and (iii) whether scholars have overlooked niche-sport systems, and whether a comparative approach holds potential to offer novel insights beyond single-sport accounts. Note that the term “niche sports” is used to define sporting systematic disciplines with smaller competitive fields and lower entry/visibility congestion/popularity. Taken together, this review structure clarifies both sport's documented benefits and persistent limits, leading to a pinpoint of which aspects SFD studies remain thin in.

### 2.1 Identity, structural inequality, and limited outcomes

The existing body of literature has long examined the empowerment of marginalized youth through sports. For instance, a follow-up study of youths in a Chilean fútbol program revealed that participants had developed a significant increase in self-confidence, teamwork, and communication skills. These carried into their daily lives, with participants noting “important changes” in personality such as overcoming shyness and becoming more vocal (13). These narratives align with the widely-used PYD lens that sport can cultivate transferable life skills as well as psychosocial resources for individual adolescents (see Section 3.1 for elaboration of theory). Furthermore, a Colombian study likewise reports that initiatives involving sports “support participants to develop new ways to think and act and to assert a sense of their capability to participate in development” (14).

However, many scholars caution that these gains sit inside larger inequalities. For instance, analyses have highlighted how although mainstream sports have potential to cultivate aspiration, they rarely guarantee escape. An example is an ethnographic study from São Paulo. The investigation showed that while SFD programs indeed shape futures (with some youth even “aspiring for professionalization within the sport sector”), the opportunities provided to participants are widely filtered through neoliberal ideals and hard structural limits. As a result, the young individuals move through “regimes of hope” against long odds (15). In practice, only a small share achieve upward mobility. As Correa candidly puts it, sport is a pathway “for the few and not the many” (13). Furthermore, various Latin American analyses highlight the structural barriers embedded in popular sport pipelines. This includes overwhelming competition and uneven access to resources like coaching, nutrition, travel, and visibility (13, 16). Notably, a Costa Rican study concludes these observations in an interesting manner: although football has yielded selective individual success stories, there has been little challenging of community-level inequalities (16).

Granted, while both sides of the debate offer detailed insight on the effect of sport participation, PYD places heavy emphasis on individual mobilization. Even (16), which approaches ideas of community-level mobilization, narrates the concept generally which requires further clarification on different levels of outcome. This study, in order to avoid conflating distinct effects, will thus explicitly separate individual-level benefits (i.e., identity, agency, confidence, which are typical of PYD claims) from the broader and less-explored structural outcomes (i.e., schooling continuity for the masses, credentials, stable work, and institutional posts explored through Bourdieu's lens). This distinction informs the paper's original analysis that follows (elaborated further in Sections 3–5).

### 2.2 An over-extended focus on popular sports

Another noticeable pattern in existing scholarship is the predominance of mainstream sports, especially football, in both programs and research. Football culturally reigns in Latin America to the point where it is frequently described as “the language” of youth (17). Unsurprisingly, many initiatives and case studies center on it. The Inter-American Development Bank's A Ganar program is a clear example: it uses soccer as a “hook” to engage at-risk youth, framing the sport as a method to teach employability skills (18). Likewise, a systematic mapping reported football to be the single most-played sport across initiatives for every continent (19). On the academic side (beyond branding), a 2016 peer-reviewed integrative study found football to be by far the most common activity in SFD-centered projects and research, especially those targeting youth (20).

As a result, other sports in Latin America aside from football are often neglected (19). found that 48.8% (*n* = 384) of the 787 organizations that specified sport type used multiple sports. This was often used to broaden appeal, scaffold different skills, and also keep doors open for varied pathways. Despite this, with peer-reviewed analyses like (19, 20) highlighting that football is placed heavy emphasis on in many SFD scholarships, other sports have rarely been explored. This imbalance is crucial: From a systematic perspective, large and highly competitive sports likely create congestion and raise capital thresholds (i.e., fees, coaching, travel, and networks). Conversely, participation in multi-sport or niche-sport activities may create altered opportunity structures due to reduced competition (more results and recognition), more access to modest but concrete resources, and beyond. This is not an *a priori* claim that niche sports are superior. Rather, it identifies a comparative and testable hypothesis that existing football-centric work has left under-specified in Latin American literature.

### 2.3 An overlooked gap

The field's narrow focus, the lack of comparison between benefits (both structural and individual) of varying sport systems, leaves an open gap for this study to investigate. I therefore aim to explore the question of whether different sporting systems can generate varying patterns of identity formation and mobility. A notable exception of the football-predominant focus is a recent study of an Olympic race-walking program in Colombia. This study tracked youth from a Bogotá community club to international competition and their takebacks from the experience, illustrates the plausibility of non-football pathways (21). Nonetheless, such scholarship remains rare. Program evaluations indicate that multi-sport initiatives aside from soccer can indeed be effective, with A Ganar reporting ∼70% graduation and ∼65% employment/education placement across countries (22). However, peer-reviewed analyses, especially ones that interrogate the systems and benefits of mainstream/niche sports in Latin America, remain heavily limited.

In essence, this literature review has shown that prior SFD research documents both (a) individual benefits and (b) structural constraints in popular sports (1–3). It also shows the empirical predominance of football in both programs and publications (18, 20). What is missing is a comparative, theory-aware test of whether smaller and less congested fields (likely found in niche sports) offer more effective forms of mobility than mass-participation fields. Expanding on concepts already used in existing literature (i.e., PYD for life-skills claims), this gap is substantive and addressable. However, as emphasized in Section 3, frameworks like PYD may not be substantial enough to explain how different sports structures work, and how they convert participation into durable educational, economic, or institutional outcomes. The review ultimately motivates the present study's comparative focus on a mainstream (football) vs. a niche (badminton) sport, and on outcomes that extend beyond immediate uplift to include education continuity, access to small stipends/scholarships, and entry into institutional roles. These are some points where opportunity converts to mobility, as will be expanded upon in the following section using additional frameworks.

## 3 Theoretical framework

### 3.1 Existing theories and frameworks

SFD scholarship has long portrayed sport as a lever for positive social outcomes. A widely cited review (14) shows a framework that dominates in such studies—Positive Youth Development (PYD). This is a theoretical lens which foregrounds life skills, character, and social capital approaches. However, PYD centers on individual change and often tends to be apolitical, as a result sidestepping the political-economic and cultural contexts that typically shape sport (23). Studies like (14) also draw on Pierre Bourdieu's fields framework, examining how sports have internal structural barriers. These lenses have produced insightful findings: PYD, for instance, showcased how programs frequently report and sustain gains in self-esteem and teamwork. Bourdieu's field-and-capital lens, on the other hand, speaks to systems and structure. Yet, it is seldom used in comparative, multi-sport analyses. The result is a literature that spotlights various micro-level benefits of sports participation, while muting how different sport-specific barriers may condition mobility differently.

Despite this, a limited number of scholars have explicitly employed personal theories and lenses to address the larger structural forces that shape sport outcomes. Notably, Spaaij's research, using Bourdieu's capital concept of “fields,” shows that mainstream sports (elite women cycling) often mirror and reinforce existing inequalities. He discovers that a youth sports program in Rio de Janeiro created new opportunities; however, the participants' “opportunities and pathways [were] not distributed evenly” was still strongly affected by structural and social factors like race, economic status, and gender (24). This indicates that any benefits were largely individualistic rather than inciting community-wide change. More broadly, Bourdieu's framework allowed Spaaij and colleagues to caution that sport often reproduces social hierarchies and perpetuates stratification.

Yet, these structurally-aware studies remain limited in scope. Specifically, they often focus on single-sport contexts, especially mainstream ones, and lack a framework to compare how individual agency and systemic forces interact across *different* sports. Context matters, but scholarship lacks a comprehensive lens to identify the sports contexts and corresponding features that best foster social mobility and lead to better outcomes. To fill this gap, this study adopts two complementary theoretical frameworks, with a heavy focus of expanding Bourdieu's theory across niche sport systems.

### 3.2 Expanded theoretical frameworks to address the gap

This study's analysis draws on two theories. First, I extend Bourdieu's theory of field, capital, and habitus across two sports. Second, I incorporate Amartya Sen's Human Capability Approach (HCA). Bourdieu's framework allows for an illumination of varying power and structure within sport systems, showcasing why different sports may offer unequal opportunities. HCA effectively centers the real freedoms people have—in other words, the ability one has to truly convert available opportunities into valued outcomes. Together, they support a holistic assessment of whether and how mainstream/niche sports' access and context shape what individuals can actually achieve.

#### 3.2.1 Bourdieu's theory of fields and capital

Bourdieu views society as something constructed of multiple “fields” (e.g., education, economy, or sport) where actors are constantly competing for status and resources. “Habitus”, defined as durable dispositions formed by social conditions, guides action in these fields (25). For instance, youth from barrios and elite academies alike will thus internalize norms that shape their behavior, aspiration, and ultimately even potential. Bourdieu also broadens the idea of “capital” beyond the economic to include social, cultural, and symbolic forms. Capital, in these aspects, include resources such as connections, institutional know-how, and recognition. These can then be accumulated and converted within and across fields. Putting this into the concept of sport, youth can, for example, earn money from advertisements and tournaments (economic capital). However, youth may also gain mentors and networks (social capital), institutional navigation skills (cultural capital), and status (symbolic capital). Bourdieu's theory of fields and capital helps explain how access to and accumulation of resources is unequal across varying systems, and why some gain opportunities easier while others struggle. The “playing field” for capital accumulation is unbalanced (25).

Scholars have applied Bourdieu's theory to education, youth, mobility, and, at times, SFD. For example (26), map sports and the SFD sector as a Bourdieusian field, analyzing stakeholder power via habitus and capital. To my knowledge, however, this lens has not been used for a comparative multi-sport analysis in Latin America. This study therefore treats each sport as a field and compares two sports to examine how power and inequality operate within and across them. It asks how distinct sport systems condition the forms of capital marginalized youth can gain, and whether participation in a niche sport yields a different capital mix than soccer. Still, though dominant in this study, Bourdieu's framework alone does not pinpoint perfectly which resources matter most for youth well-being and opportunities; it is here that a second framework is used.

#### 3.2.2 Sen's human capability approach

To complement Bourdieu and enhance the study's findings, I incorporate Sen's Human Capability Approach (HCA) into my analysis. HCA is a people-centered framework that shifts attention from means to ends. In other words, it explores the possible long-term effects connected to the activity, starting from participation to what individuals are substantively free to do and be. Notably, HCA distinguishes among *resources*, *capabilities* (real opportunities), and *functionings* (achieved beings/doings). Applied to sport, the question is therefore not only “Did a job result?” It can now be expanded to “What valued options can participation open, whether it is education, work, health, or social ties, and with what depth and durability?” HCA thus frames success as expanded meaningful choice, assisting to capture outcomes beyond narrow economic mobility (12).

Although rooted in development economics and ethics, HCA has only recently entered SFD research. Pioneering works like (27) apply HCA to sport programs, and calls persist for further studies grounded in development theory (including HCA). In this study specifically, I integrate HCA across the two sporting cases to evaluate the varying capabilities and functionings stemming from different sports. I thereby test whether alternative sport types have the potential to yield distinct long-term mobility outcomes.

#### 3.2.3 Field–capability theory integration: analytical strategy and operationalization

Taken together, these frameworks provide a single, complementary logic for the study. Bourdieu's concept of *field* helps to clarify why opportunity structures differ across sports; the “field of struggles” in which positions and forms of capital (power) are contested and unequally distributed (28). Sen's Capability Approach specifies *how* those endowments are (or are not) converted into valued *functionings*, the “freedom to achieve well-being” shaped by context-dependent conversion factors (12). I operationalize the integration of these two frameworks in three steps: (i) map sport-specific capitals and thresholds in soccer's congested field vs. badminton's smaller, more permeable one; then (ii) evaluate the conversion environment (i.e., institutional recognition, mentorship access, time compatibility with schooling, and stipend/scholarship routes) as factors enabling or blocking capabilities and outcomes in the triangulated interviews and documents; and finally (iii) include a section analyzing the field (systematic structure) of each sport and connecting that to why selection effects arise and opportunity thresholds differ across sports. This process yields two distinct bridges. First, a causal (why) bridge (field-based distribution and selection effects), and second, an analytical (how) bridge (capability conversion). With the empirical gap primarily revolving around football-dominated studies, single-sport designs, and the need to test whether niche sports alter mobility pathways, this structure–agency integration serves as the most suitable framework. Specifically, this integration process links sport-specific opportunity structures to the mechanisms by which participation does, or does not, become durable mobility. Especially considering the limited statistical data for niche sports available to the study (see Section 4.3), this dual framework allows for a strengthened interpretation and thus more confidence in our analysis. This enables a systematic investigation of potential differences across sports, directly addressing the highlighted gap.

## 4 Methodology

This study uses a multi-sport case design, examining how structural barriers and youth mobility outcomes may differ between a mainstream and niche sport within Latin America. By examining and building on research regarding sport and social exclusion, I analyze two cases (soccer as mainstream and badminton as niche) through Bourdieu's and Sen's frameworks. This novel comparison tests whether a less popular sport can open pathways that a dominant sport does not. Data-wise, I triangulate quantitative and qualitative evidence to trace how barriers manifest differently in each sport and where mobility opportunities emerge.

### 4.1 Case selection and sampling

The region (Latin America) is kept constant, although data occasionally references other regions for comparative purposes. I selected soccer and badminton to differentiate between a widely played sport and a niche one.

#### 4.1.1 Soccer (mainstream)

Soccer is the region's dominant sport (29), providing a natural and relevant baseline. Prior studies show that structural barriers constrain youth advancement even in mainstream sports like soccer. Using the aforementioned frameworks, I extend this evidence, treating regional soccer as the benchmark for mobility outcomes typically achievable in the most established sport. I then compare the results to badminton (see below).

#### 4.1.2 Badminton (niche)

Badminton remains relatively obscure in Latin America despite its Olympic presence. Institutional support for the sport, however, has grown in recent decades. For instance, a regional youth program reported a 71.6% rise in participants [though total participants was below 4,000 (30)]. Although empirical data remains limited (see Section 4.2, 4.3), badminton's emerging popularity provides the study substantial evidence to initiate research through this lens (31). This allows for a test of whether less popular sports, with smaller competitive fields and different institutions, face fewer barriers or simply different ones. Badminton is analyzed as the comparative case to soccer.

### 4.2 Data sources

#### 4.2.1 Data corpus

The core data corpus is assembled using 47 media sources. Language in sources vary across English, Spanish, and Portuguese. Sources range across three types: (i) peer-reviewed journal articles and analyses; (ii) program/policy evaluations which are sourced from federation or government reports; and (iii) and reputable long-form media, including but not limited to documented primary interviews with athletes and encyclopedia entries. Searches were last updated September 2025 throughout revision, and were run in Web of Science, Scopus, Google Scholar, and policy/sport portals (i.e., the Inter-American Development Bank; FIFA; Badminton World Federation; national Olympic committees; national sport ministries and federations). The core strings combined sport × region × outcome terms. For example:

“Latin America AND (soccer OR football) AND (education OR employment OR mobility)”;

“badminton AND [Latin America OR (country)] AND (youth OR pathway OR stipend).”

Inclusion: substantive discussion of youth pathways, resources, employment/education, or earnings in soccer/badminton; Latin America/Caribbean focus; original analysis, statistics, or interviews. Exclusion: match recaps, syndications/duplicates, pieces without mobility/structure content.

Source hierarchy & translation checks: Under uneven evidence (*see below*), I privileged peer-reviewed and official sources over program/press. Furthermore, I privileged program/press over general media. Non-English content was translated and furthermore spot-checked by a bilingual reviewer. When multiple sources addressed the same claim, convergent evidence was prioritized.

#### 4.2.2 Primary interviews

In combination with our core data corpus (Section 4.2.1), three semi-structured key-informant interviews (with Kevin Cordón, Inés Castillo, Taymara Oropesa) were conducted along with a native Spanish speaker. All three informed interpretation. Our interview with Guatemalan Olympian Kevin Cordón (July 11, in-person, ∼15.5 min) was used specifically in the study, as the other two echoed the same themes and are therefore summarized rather than quoted. The topics covered included the level of access barriers, their views towards the sport, institutional support, capital conversion, and post-sport options (see Table 1), with questions five to eight being especially important. Audio was recorded with consent of all participants; it was transcribed verbatim, and translated from Spanish to English with bilingual verification from a native speaker. The participants were informed that they could stop the interview whenever desired.

**Table 1:** Semi-structured interview with Kevin Cordón: verbatim question guide and timestamps. Questions 5–8 (why Cordón chose badminton and how participation has shaped his development and mobility) are especially pertinent to the study's SFD-through-niche-sports focus.

Q.	Question	Time Stamps
1	Kevin, please tell us about yourself. Can you tell us how you were introduced to badminton? Where are you from and how old are you?	1:10–2:12
2	You weren't born into a badminton legacy, and instead you discovered it, almost in a way by accident growing up in La Unión, Zacapa. Can you take us back to your early training environment? What did facilities look like in your town?	2:12–3:51
3	Latin America generally struggles to secure major international badminton titles compared to Asia or Europe. Why do you think this gap exists? What are the main obstacles for Latin American athletes?	3:52–5:18
4	Building on the few questions before this one, if you were to list 2–3 important things that would boost your chances of receiving an Olympic medal, what would they be?	5:19–6:33
5	Did badminton give you a better chance of becoming an Olympian than if you played a different sport? Would you say climbing that Olympic ladder was comparatively easier because there were less people competing? Or did playing a slightly less popular sport give you certain advantages?	6:35–8:37
6	And at the end of the day, what did becoming an olympian and continuing badminton mean for you?	8:40–9:46
7	We know that Guatemala is known for football popularity. If you played an even more competitive sport like fútbol, would your opportunities perhaps be different? Or baseball? What's better about sports like badminton for your development?	9:49–10:50
8	Some say sport is for a healthy body. Others say it's simply for earning money. If you were to describe what sport means to you in 3–4 sentences, what would you say?	10:52–12:18
9	To all listening in Guatemala and the Pan Am region, whether it's competitive players or young children aspiring to play badminton, what advice would you offer them?	12: 20–13:58
10	What role do you believe athletes themselves can play in promoting social change through sports? Do you think it is possible for the Pan Am region to share the global stage with the world?	14:00–15:12

Given the small-N, interviews are treated as illustrative mechanism evidence. Nonetheless, if corroborated with documentary/indicator data, the relevant findings are treated as robust or suggestive rather than illustrative in the Results and Discussion (see Section 4.3).

#### 4.2.3 Quantitative indicators

I compiled eight indicators to contextualize opportunity structures and obtained results across sports. The indicators, along with their value to the study, are listed in Table 2 (not necessarily in order). All the monetary amounts reported were left in nominal currency (US$). This avoids spurious precision across heterogeneous sources. Regarding the indicators, archives thus far unfortunately contain limited data for most niche sports. Not all indicators were available for badminton (i.e., player salary distributions, sport-economy shares), so further research should aim to compile these statistics for expanded investigation (*see below*).

**Table 2:** Quantitative indicators used (when possible and appropriate) to contextualize opportunity structures across sports.

Indicator	Relevance to the study
1. Membership/club density	Shows how widely each sport is practiced and how accessible organized participation is.
2. Stipend/scholarship coverage (e.g., Bolsa Atleta, Olympic Solidarity)	Indicates the extent of financial and institutional support available to athletes. Examples include Bolsa Atleta, Olympic Solidarity, etc.
3. Player salary distributions	Provides a sense of how earnings and professional rewards differ across sports. For instance, FIFPRO reports (limited for badminton)
4. Sport revenues/prize money (FIFA, BWF annuals 2007–2023)	Reflects the overall financial scale and commercial reach of each sport. Example sources include FIFA BWF annuals, etc.
5. National sport-economy shares (where available)	Suggests how much attention or investment each sport receives within national contexts.
6. World-ranking penetration (Pan Am/World Rankings)	Observes how athletes reach competitive international levels and their rate of progression.
7. Participation trends (e.g., youth program growth)	Helps identify whether engagement in the sport is expanding or declining, showing the network athletes find themselves in (and also the competitiveness of the system).
8. Institutional team posts (military, police, club squads)	Points to stable career options that allow athletes to stay involved in sport and networks after competition.

### 4.3 Analytical strategy

This study uses a comparative case study approach, where each distinct sport is analyzed through a common framework and then examined together. For each case, I first outlined the sport's context. Then, I identified key structural barriers, assessed economic outcomes, and analyzed social/cultural outcomes (focusing on evidence of athlete mobility and long-term translations using Sen's framework). Finally, I interpreted these findings through Bourdieu's “fields” lens, examining why differentiating opportunities may arise (as noted in Section 3.2.3). Qualitative data from our interviews along with personal narratives from our data corpus were synthesized into these thematic categories and presented in conjunction with quantitative data to contextualize the patterns observed.

It is important to note that data availability is uneven across selected cases. Soccer has been widely explored, and is richly documented in multiple peer-reviewed studies and union surveys Furthermore, official statistics for nearly all of the eight aforementioned indicators exist. Information about badminton and niche sports, on the other hand, are less present in studies, and only documented in program documents, federation/ministerial materials, reputable long-form media, and athlete/coaching narratives. To manage this imbalance, I: (i) triangulate across different source types to enhance confidence; (ii) distinguish between secured patterns and single-narrative accounts; and (iii) signal the level of confidence in claims by noting whether they are illustrative, suggestive, or robust in the Results and Discussion sections. See Table 3 for how the claims were sorted into these three confidence hierarchies, indicating which parts of the study were presented with increased or decreased confidence. As mentioned previously, claims for soccer are typically stronger on quantitative indicators. Badminton conclusions are typically bound to mechanism-level inferences where indicators are sparse. Accordingly, findings about badminton and niche sports' potential contribution to broader structural change are often reported as illustrative mechanisms rather than definitive outcomes (unless corroborated with peer-reviewed data). Evidence on more immediate access, visibility, and pathway opportunities are conversely reported with high confidence. Crucially, this approach is designed to provide cautious but reliable leverage on an under-researched area. While the asymmetry of evidence constrains generalization (mitigated through the regional literature base of eight different countries), the comparative framework and confidence hierarchy assists in ensuring that reported patterns are credible enough to motivate further investigation of niche sports in Latin America.

**Table 3:** Triangulation confidence hierarchy. This table highlights how each of the three selected confidence levels are determined, what they establish/indicate, and how they are signaled in-text.

Confidence Level	What it Establishes	Constituting Criteria	In-Text Indicators
Robust	A pattern that is appropriate for systematic statements due to supporting evidence across multiple sources and narratives. These claims are interpreted as general features of the sport system with the stated scope.	Information is treated as “Robust” when convergent evidence is provided from multiple (≥2) rigorous sources like peer-reviewed articles and official policy documents. There is also increased confidence when quantitative indicators align with qualitative accounts and the two theoretical frameworks (Bourdieu/Sen). Any interview information used is corroborative and is not taken as “Robust” on its own.	High confidence findings are indicated through phrases such as “well-documented” or “there is increased confidence […]”.
Suggestive	A plausible trend that is consistent with theory and some data. However, there is not yet substantial corroboration or confirmation from other sources. Though offering substantial confidence, information should be read as directional evidence and not conclusive.	Information is treated as “Suggestive” when it comes from a single rigorous source but not multiple. A claim can also be “Suggestive” if there is partial coverage (e.g., information about one country/program) or limited statistics. Requires additional corroboration to be treated as systematic.	Plausible trends are indicated with phrases such as “suggests that” or “likely”.
Illustrative	A mechanism-level example that clarifies narratives or processes for specific individuals/contexts. These results should be treated as informative but not as a prevalence estimate.	If not corroborated with rigorous sources, selective narratives and interviews (small N) are treated as “Illustrative”. These trends and information may align with theory but lack the substantive quantitative indicators and/or qualitative analyses to confirm directionality or breadth.	These areas with lower confidence are flagged with indicators such as “illustrative” and “mechanism-level”.

## 5 Results and findings

### 5.1 Soccer—rewarding or precarious?

Soccer, played across social strata, is by far the most popular sport in Latin America. Its influence has been noted to “transcends class, race, religion, gender and educational background” (32), making it appear accessible to anyone with talent. Indeed, Latin American nations have excelled on the world stage. However, prior scholarship shows limited broad benefit for the majority of athletes. This paradox frames the questions of this case: does soccer truly promote social mobility for youth in Latin America? Or does it offer hope to many but prosperity to only a few?

#### 5.1.1 Busting the myths

From the barrios of Buenos Aires to the favelas of Rio de Janeiro, millions of youths dream of becoming professional footballers. However, hard data reveals how extraordinarily slim those chances are. Out of every 10,000 Latin American players, only one will ever sign a professional club contract (roughly 0.01%) (33). Even in Brazil's prolific talent pipelines, the chances of success are approximately one in 3,000 (33). Amidst this, however, there have been notable success stories; indeed, it is commonly said in Brazil that “for the poor, especially the dark-skinned poor, social mobility can come only through soccer, music, or drug trafficking” (34). The media's idolization of rags-to-riches stars such as Messi, Pelé, and Maradona perpetuates this folkloric belief. Yet for every poor kid who *does* make it big, millions more have chased the same dream and fallen short.

In addition, soccer too often becomes a substitute for schooling rather than a parallel track. When “soccer is all I am,” outcomes are bleak for those excluded—skills and credentials lag, human capital is thin, and reentry into education or work becomes progressively harder (35). In Sen's terms, athletes' capabilities shrink as structural pressures push these poor youth to risk everything on one precarious path. Note that prioritizing sport, at many times, is appropriate. It is soccer's “precarious” structure that makes that bet especially risky.

#### 5.1.2 Precarious careers and limited mobility outcomes

##### 5.1.2.1. Economic mobility

For the minority who become professionals, systemic features of the soccer field sharply limit upward mobility. Union surveys indicate that globally fewer than 2% of players earn >$720,000 per year, while about 45% earn <$1,000 per month; comparable or harsher distributions characterize many Latin American leagues (36). Pattern-level historical data from Brazil found that only 4.3% of professionals earning was greater than US $1,350 per month in the late 1990s, while 83.4% earned less than a shocking $120/month (36). Region-wide, the sports industry accounts for ≈1% of GDP (vs. ≈4% in the U.S.). This suggests limited spillovers into broad employment (33). Reading through Bourdieu's field logic, a congested field concentrates economic rewards at the top and shortens careers for most. In Sen's terms, the post-sport functionings (such as stable employment or economic security) available to a lower-division player are often quite limited. Most retired players, lacking savings and formal education, may end up only marginally better off than if he had never played the sport—perhaps even worse off for having foregone other opportunities. With the triangulation of aforementioned peer review analyses with multi-source indicators and theoretical frameworks, there is confidence that soccer's contribution to sustained economic mobility for most participants is disappointingly limited. This prompts the question: Does soccer offer other benefits for social mobility?

##### 5.1.2.2. Social and cultural

Given that most Latin American youths who pursue soccer never truly secure a lucrative professional career, any chance for upward mobility must then come from intangible gains. In Bourdieu's terms, these take the form of social or cultural capital, including networks, education, and beyond. However, this study finds that the idea of soccer as a great social equalizer is as tenuous as its economic promise. Research has dispelled the notion that sports automatically levels the playing field. In fact, as mentioned previously, opportunities and outcomes are heavily stratified by class and other factors, an observation that holds especially true in Latin American soccer. For instance, the sport's intense competitiveness and the time commitment required to reach the top help explain why more than 35% of professional Argentinian fútbol players are high school dropouts. In 2023, former football star Carlos Tevez revealed that many players in his top-division squad “did not know how to add or subtract,” highlighting how the time-commitment and single-minded focus on fútbol can come at the expense of basic schooling (37). Argentinian programs today sponsor schooling for roughly 3,000 players per year. Experts, however, say about 30,000 are needed (37). In short, chasing a soccer career often isolates young athletes from mainstream educational development.

Aside from the realm of education, proponents often highlight that participation in soccer builds immense confidence and social skills that are vital to one's progression. Some youths indeed do gain self-esteem and teamwork skills through sports. For many aspiring players who pin their identity entirely on soccer, however, the difficulty of the career process can trigger a significant loss of self-worth and social confidence. Carlos Acevedo, a Mexican goalkeeper, blatantly illustrates this toll. In a 2022 interview, he explained that by dedicating himself to football from age 10, he lost the opportunity to experience normal adolescence which left him socially isolated:

“Yo desde muy chico estoy ligado a lo que es el deporte, desde los 10 años, entonces todo ese proceso de borracheras, de 15 años, yo no lo viví porque yo estaba entrenando, estaba de viaje … Me quedé sin amigos, me quedé sin nada, todo por el fútbol. Obviamente que no me arrepiento, pero me hubiera encantado vivir esa adolescencia normal que tiene un junior o un mirrey.”

—Mexican professional footballer Carlos Acevedo López (born 1996), reflecting on his youth career in an interview with *Récord* (38).

Francisca Moroso, a former Chilean national team forward who retired in 2021, similarly portrays the social downsides of soccer participation through citing her depression. She experienced many positive moments in her career, but the toxic environment (specifically abuse from coaches) ultimately shattered her self-esteem. In her retirement announcement, Moroso wrote that “Cuando las cosas ya no te llenan el alma y no te sientes con las energías para entregarlo todo (porque una persona cambió el destino de tu carrera y te hizo sentir minúscula), es momento de detenerse.” She further remarked that “depresión es una enfermedad terrible que arrasa con todo” and declared, “Hoy dejo el fútbol para jugar otro partido, el más importante de mi vida” (39).

Their experiences reveal the hidden emotional toll of a soccer pursuit. These stories and statistics illustrate that the competitive sport's promised social (and economic) uplift is far from guaranteed. In addition, even a successful career can come at the expense of one's social life and mental health. Seen through Sen's lens, symbolic recognition and advancement accrue to a very small fraction; for many, identity investments in soccer alongside the erosion of education do not reliably convert to durable capabilities (credentials, recognized skills). Instead, there is a heavy erosion of wellbeing when advancement stalls. This in Sen's terms represents a capability “deprivation”, where the loss is not only pertaining to immediate economic prospects but also of the substantive freedoms to pursue long-term stability and/or self-defined wellbeing beyond the sport itself.

This study now adopts Bourdieu's social capital and fields analysis to examine the potential reasons behind this phenomenon.

#### 5.1.3 Structural barriers and capital in the “field” of soccer

Bourdieu's theory aids in explaining why the promise of meritocratic ascent so often falls short. As he notes, “class habitus defines the meaning conferred on sporting activity [and] the profits expected from it.” (40) For many working-class and marginalized youth in Latin America, soccer is a high-stakes investment. The profit sought is life-changing mobility, not just recreation or health. Yet, success in the soccer field requires more than talent. It hinges on capital that is uneven, and more crucially, is largely beyond youths' control. Higher demands for mainstream sports include increased need for economic capital (fees, travel, nutrition, specialized coaching), social capital (networks, agents, coaches), and cultural capital (know-how to navigate elite institutions) are often not up to the athlete to decide. This pattern holds true in various global regions, revealing a general pattern. A study in Spain, mirroring patterns in Argentina and Brazil, found that players who relocated from small towns to urban centers with stronger clubs had 38% higher odds of debuting professionally (41).

Put simply, proximity to privileged hubs brings better coaching, exposure, and competition. Families with the means or supportive networks to move accumulate “sporting capital” that sharply boosts prospects. Equally talented youth on the other hand have a higher chance to remain in disadvantaged settings and drop out or become overlooked. In Bourdieu's terms, the soccer field operates by a logic of capital. It rewards the strategic accumulation of resources and ties more than raw ability. As a result, geographic, economic, and racial inequalities shape who reaches the top, reinforcing the original inequalities facing communities. For Latin America specifically, a study in Brazil by Francisco Rodrigues shows that “soccer mirrors regional dissimilarities”. The North, Midwest, and Northeast have the least-developed markets, poorest conditions, lowest wages, and greatest vulnerability, while the Southeast and South offer substantially higher pay and stability (42). Even insiders like coaches recognize these biases. Veteran coach Givanildo Oliveira, coming from Northeast Brazil, noted furiously during an interview that “prejudice does exist … Given everything I've won, everything I've done and am still doing, I'm certain that if I weren't from there [the Northeast], I'd already be coaching a top team.” (43) Fittingly, barely 10% of Brazilian World Cup players come entirely from the Northeast, supporting this idea of intra-sport inequalities (44).

The convergence of peer-reviewed statistics in various regional studies alongside personal narratives lends robust confidence to these findings. This, together with analysis using Bourdieu's/Sen's frameworks and existing scholarships, further highlight that persistent inequality indeed exists within soccer's structural system (and potentially other “elite” sports). Yet, does this pattern of structural limitation hold true across all sports? Could less-popular sports potentially offer more equitable pathways for genuine mobility?

### 5.2 Badminton: a potential unsung Hero for mobility

Evidence note: As detailed in the Methods, although the study aims to triangulate observations whenever possible, empirical badminton evidence (i.e., graduation rates, annual salaries, etc.) is thinner and more case-based than for football; findings are indicated and presented as suggestive or illustrative unless policy or multi-source indicators permit a robust inference.

This study investigates whether, in Latin America's football-dominated landscape, a niche sport such as badminton can offer a more accessible route to athletic success and social mobility. Some athletes make this calculation explicitly, choosing less crowded fields to be seen. Guatemalan Olympic badminton player Cordón is a prime example; as a boy from a marginalized community, he realized that badminton was not popular in his soccer-crazy country and believed he would have a better chance of standing out in that field. He was right—moving from a remote, resource-poor town to four Olympic Games and national icon status in a sport many compatriots once struggled to pronounce. Parallel trajectories (Brazil's Ygor Coelho; El Salvador's Uriel Canjura) point to the same mechanism: niche sports can open distinct opportunities, even if the economic upside is thinner than football's. Read through Sen's lens, these paths expand capabilities—the real options to study, travel, and access institutional roles—precisely because, as Bourdieu would note, the smaller field lowers capital thresholds and accelerates symbolic recognition, making conversion more attainable. Regionally, policy scaffolding in Mexico, Paraguay, and beyond (see Section 5.2.1.1) further supports conversion for Olympic pathway athletes, shown in documented official programs which enhance confidence. These programs connect recognition in smaller, less congested sports to predictable material support such as stipends, scholarships, and study flexibility, turning symbolic achievement into tangible mobility outcomes. In effect, this results section demonstrates how the institutional architecture of niche sports holds potential to transform early success into long-term capability gains, even when absolute financial returns remain limited.

#### 5.2.1 Higher chances through smaller gains

##### 5.2.1.1 Economic capital: opportunities and trade-offs in niche sports

Choosing a niche sport may be a deliberate way to move up faster on the social mobility ladder, though it comes with noticeable trade-offs. In settings where football absorbs most attention and resources, a talented badminton player may see doors opening simply because there are fewer competitors. Cordón, for instance, recalls that as a teenager “[his] whole family loved football … but when [he] saw that badminton was opening doors for [him] … [he] decided to dedicate [him]self to it” (45). However, badminton opened opportunities he could act on. Within three months of picking up a racket he won a local tournament. By his mid-20s he had “surged to [world] number 29”, won two Pan American championships; these are milestones far harder to reach in more congested sports. Similarly, Brazil's Coelho rose from a favela court to reach world No. 51 by the age of 25 (46). Seen through Bourdieu's framework, a thinner field speeds symbolic capital accumulation (national titles, international caps, elaborated in Section 5.2.3), which Sen would evaluate as capability gains—more plausible access to support, travel, institutional posts, and recognition with fame.

Despite this, this study acknowledges that badminton's financial rewards remain far more limited than soccer. Badminton lacks the lucrative salaries and sponsorships that top footballers enjoy; for context, the Badminton World Federation's total gross income in 2023 was about US$43 million, a tiny sum next to FIFA's revenue of around US$1.17 billion the same year (47, 48). Although global prize money in badminton has grown (from ∼US$4M in 2007 to ∼US$18M in 2023), it “remains low compared to other sports.” (49) Latin American badminton players often rely on modest stipends from government programs or Olympic Solidarity grants rather than professional contracts. As Cordón candidly highlighted during his Tokyo Olympic run: “This, for us (Guatemalans), is not easy. We have to work, we still have to fight, we need some more support.” (49) His longtime coach, José María Solís, has likewise urged Guatemalan authorities to invest more in sports such as badminton, noting that disciplines such as badminton, racewalking, and gymnastics are “doing a lot for Guatemala” on the world stage and deserve greater funding (45). In essence, it may be easier to become a big fish in the small pond of badminton, but that pond holds far fewer financial resources than the ocean of football.

Nevertheless, amidst these challenges, achieving success in niche sports can still yield economic benefits or stability. National Olympic committees often provide stipends to Olympians, and medalists earn government bonuses. Even on a smaller scale, being a top badminton player in a Latin American country can lead to jobs within sports federations, paid coaching roles, or scholarships to study and train abroad—forms of economic capital that would be inaccessible without the athlete's niche success. For instance, the study's extensive regional investigation found that Mexico's CONADE administers Becas Económicas (athletic scholarships) for elite athletes (including application portals) (7, 50); the International Olympic Committee's Olympic Solidarity provides direct athlete scholarships for qualification cycles (51); Brazil's Bolsa Atleta (2005–) is one of the world's largest direct stipend schemes and has supported badminton athletes including Coelho and Lohaynny Vicente (52, 53); Paraguay's SND allocates monthly support, travel and insurance to high-performance athletes per official resolutions (54); Peru's IPD/VIDENA hosts BWF events and, together with universities, grants “Deportista Calificado” scholarships that formalize study–training balance and economic support to athletes (55, 56); and finally, Ecuador's Plan de Alto Rendimiento couples training with education flexibility and scholarships for selected athletes (55). Viewed through Sen's capability framework, these state and Olympic programs not only compensate for the smaller economic scale of niche sports, but also convert success in a less competitive field into meaningful *functionings*. This includes the ability to pursue education, secure steady income, and participate fully in social life. In doing so, they reposition badminton not merely as athletic success, but as a credible pathway toward incremental social mobility within resource-constrained contexts.

Moreover, in some Latin American countries, successful badminton players find stability by joining institutional teams (e.g., sports clubs or military squads). In Brazil and Ecuador for instance, it is common for athletes in niche sports to enlist in the Army or Air Force which have “athlete-soldier” programs providing a salary and access to training facilities. A notable example is Lohaynny Vicente, a successful female player from a Rio de Janeiro favela, who is employed by the Brazilian Air Force. This has given her a monthly salary to compete for their sports division and also helped her family move into a new apartment and attain a middle-class lifestyle (56). These opportunities, while not the most lucrative, offer a distinct contrast to the limited local job options most had before.

In summary, the economic equation of pursuing a niche sport in Latin America involves lower (yet beneficial) absolute rewards but a higher probability of accessing them compared to the long-shot odds of achieving football stardom. As illustrated by these narratives, the selection of a niche sport can “open doors” to international travel, education, and modest professional livelihoods that might have remained closed on the far more congested road of mainstream sports. However, economic capital is not the only source of mobility in Bourdieu's and Sen's lens. The next section delves into social and cultural capital gained from badminton athletes, some of which have been found to yield even more compelling results than the economic benefits.

#### 5.2.2 Social and cultural capital

Beyond financial gain, participation in badminton also exposes athletes to new cultures, networks, and life skills. Kevin Cordón's Olympic odyssey “changed [his] life completely”, transforming a small-town boy into a worldly and mature individual. Because of badminton, he was able to travel globally and gain chances to speak on international platforms alongside facing the triumphs and trials that forged resilience. “Sport is like life, there are highs and lows,” Cordón reflected. He notes that by overcoming challenges in competition, he became “a different, more mature, more conscious person.”^1^ Read through Sen's lens, this is capability expansion. Specifically, it is the growth in the substantive freedoms to communicate, to travel, and to pursue self-defined wellbeing. Bourdieu's social capital theory simultaneously recognizes the accumulation of embodied cultural capital (poise, discipline) with value beyond sport.

This principle exists beyond individual and star athletes into regional communities. Sebastião Dias de Oliveira, the founder of the renowned Miratus badminton project in Rio's Chacrinha favela, deliberately started badminton training as a counterweight to gang recruitment. “A ideia foi fazer frente ao crime organizado através de uma peteca e uma raquete,” he says. De Oliveira explains his strategy bluntly:

“Nós temos que acolher essas crianças com a mesma eficiência que o crime organizado faz. Eu tive que chegar antes. Com oito para nove anos, quando o crime organizado chega, os meus garotos já estão campeões, trilhando um caminho e sendo referência para outros.” (57)

In other words, engaging children in badminton from an early age ensures that the cartel recruiters arrive too late. Through participation in badminton, the kids have already found purpose, consciousness, and positive identity. In Sen's lens, this badminton initiative specifically expands children's capabilities by offering real alternatives to crime and violence, thus allowing them to pursue meaningful and valued life outcomes that would otherwise remain out of reach due to structural marginalization. It is important to note that although Cordon's and de Oliveira highlight distinct benefits of badminton participation (individual vs. community), they are both anecdotal narratives and therefore are treated as illustrative in this study. However, along with the following narratives, there is increased confidence as analogous trajectories recur across countries. Furthermore, independent primary accounts align with documented school-based and inclusive badminton expansions. This indicates a mechanism that plausibly generalizes beyond isolated cases.

Success stories from once-marginalized athletes hold inspirational power that not only generates social and cultural capital for athletes themselves but for the communities around them. In Brazil, Coelho's story, a boy who “grew up avoiding bullets in his Rio favela” and found salvation in badminton, has resonated deeply with his environment. “Many of [Ygor's] friends died or got mixed up in drugs, but Coelho persevered and became Brazil's first-ever Olympian in badminton,” recounts one profile of him. Now, Coelho's success is “inspiring Brazilians to pick up a racquet and try the sport themselves.” He proudly observes how “a lot of kids … [are] very motivated to play,” because “they see that even though [he] came from the favelas, [he] can achieve this dream” (46). This is a powerful form of symbolic capital, where such achievements, coming from a marginalized impoverished individual, represent hope and possibility that challenge social prejudices about who can succeed. In Bourdieu's terms, Coelho has also accumulated *social* capital for his community: a tight network of admirers, protégé athletes, and institutional supporters rallying behind badminton. This accumulation of capital directly enhances his own social mobility as well by elevating his status, providing influential connections, and creating opportunities such as national recognition, leadership roles, and platforms for advocacy.

Likewise, El Salvador's Uriel Canjura, who started badminton barefoot on a dirt court, became his country's first Olympic badminton player in 2024. Canjura strongly emphasizes that in El Salvador, “football is the first sport … badminton isn't so popular in Latin America.” Yet, he acknowledges that he fell in love with badminton, and it propelled him to heights he “never imagined”. For Canjura, seeing Cordón excel on the Olympic stage was a major turning point, noting in an interview: “He's the big player for all of the Americas … a semifinal at the Olympics isn't easy, but he did that, so why can't I?” (58) Such testimonials emphasize how one athlete's cultural capital (i.e., prestige, status, and expertise) can be transmitted to others as inspiration, altering what young people perceive as attainable. In regions where social mobility pathways are limited, these sport heroes embody an alternative script for success based on merit, hard work, and passion.

On the mental side, players frequently report gains in discipline, self-esteem, and social connection. This sharply contrasts soccer where multiple accounts (including López and Moroso) describe a competitive field that can be toxic and exclusionary. A Spanish-language badminton site in Mexico notes the sport teaches “competencia justa” (fair play), “respeto mutuo” (mutual respect), and perseverance, shaping youths' identity and confidence beyond the court (59). Coaches likewise observe shy children opening up in badminton groups, gaining friends and communication skills. In the Special Olympics' Latin American badminton outreach, journalists report growing access to equipment, coaching, and training; participants such as Paraguay's Thanya López say these opportunities “help[ed] [her] socialise more, especially when [she] get[s] asked ‘what is badminton?’” Similarly, Chile's Francisca Guadalupe says badminton helps her stay “healthy” and proud to “represent [her] country and [her] school,” while meeting new friends (60). Notably, these athletes are neither national elites nor Olympians. Yet badminton's accessibility has brought them to spaces where they connect positively with others, strengthening confidence, identity, and social well-being. In Sen's terms, these enhanced social skills represent expanded capabilities, enabling athletes to pursue broader educational, professional, and social opportunities.

Continental events further normalize both badminton's presence and impact. Lima, for instance, hosted the Pan Am Individual Championships 2025 which consisted of 124 players from 16 countries (ARG, BRA, COL, CRC, DOM, ESA, GUA, GUY, MEX, PAR, PER, TTO, USA, VEN). This expands regional symbolic and social capital through institutional contacts, whether it is coaches, federations, or university partners. By raising visibility across multiple member associations, such events increase the chances that youth participants encounter education and social-networking opportunities (e.g., university placement, tutor networks) tied to national programs highlighted in Section 5.2.1.1. Networking enables participants to use what Sen terms social conversion factors—“factors from the society in which one lives” (27). These factors consist of mentors, institutions, and norms, so that shared contacts and knowledge can translate into access to education and employment. Furthermore, they echo Bourdieu's idea of social capital as “the aggregate of the actual or potential resources linked to a durable network of relationships of mutual acquaintance and recognition” (11). The participation data, coming directly from Badminton Pan America's report, presents itself as robust and provides the study with substantial confidence. However, the interpretations are still presented as suggestive, as data for post-event outcomes (i.e., athletes' subsequent access to scholarships, university placements, or continued networking with relations) remains unavailable. I encourage this data to be compiled as part of future research.

Finally, it is worth noting that most of the aforementioned players, though successful in gaining capital from sport, attended high school. Compared to soccer, badminton's less competitive field has allowed athletes to continue pursuing academics while training; Coelho, for example, “studied in a very good school in Brazil” and at a point in his career even “concentrated on studies and not on sport.” (61) Nonetheless, he still achieved major success, a result primarily attributed towards the sparser, less competitive field of badminton (see Section 5.2.3). However, quantitative data regarding school participation and scholarship rates similarly remain limited for badminton athletes. Further research could investigate statistics to expand this study's niche-sport perspective; this will better illustrate with empirical evidence how less popular sports enable athletes to balance academics with athletics and build educational capital alongside their athletic success.

#### 5.2.3 The field of badminton

Bourdieu's notion of “fields” can provide insight into the existence and significance of niche sports pathways. In Latin America, the football field is highly developed and crowded, teeming with aspiring players, massive investment, and a rigid hierarchy of talent. Conversely, the badminton field is relatively sparse and under-resourced in this region, which creates a different dynamic. For the studied players, entering the badminton field allowed them to rapidly accumulate capital in terms of skills, coaching support, grants, stipends, and titles because the initial competition was low. Cordon noted that reaching the Olympics was a more tangible goal for him when he started, given fewer competitors, though competition is gradually growing larger as the field expands.^1^ This supports the strategy of choosing a less popular sport as an “alternative pathway”: the field's structure afforded Cordón a chance to excel nationally and regionally, converting his habitus of discipline and athletic talent into tangible success without being overshadowed by thousands of rivals.

However, Bourdieu cautions us that a field confers value on capital only relative to its size. Latin America's badminton field is a small pond, and being a “big fish” there does not equate to global dominance. When Cordón stepped onto the world stage, he faced the might of Asia and Europe, fields with far greater badminton capital (coaching expertise, player base, and generous funding). Despite his regional supremacy, Cordón had to struggle against structural disadvantages, such as limited sparring partners and lack of high-performance science,^1^ which are abundant in countries where badminton is a major sport. Nevertheless, within this uneven playing field, becoming a top player in Latin America brings substantial benefits already. Moreover, the gap is slowly closing; “Asian powerhouses face a tougher challenge from Europeans, Latin Americans and North Americans than they have in the past,” reported Reuters during the 2024 Olympics, highlighting a notable shift (47). The field of badminton is expanding and progressively democratizing, which may improve the prospects (and rewards) for Latin American athletes in the long run.

## 6 Discussion

The multi-sport study analyzed a popular vs. niche sport, highlighting their distinct effects on social mobility outcomes for marginalized youth in Latin America. While soccer indeed is culturally dominant, its capacity to deliver broad social mobility is found to be both economically and socially limited. Intense competition and structural inequalities result in only a tiny fraction of aspiring players, usually those already advantaged, attaining elite professional status. This illustrates how soccer's internally uneven framework can strongly perpetuate existing socioeconomic disparities. In contrast, the niche sport badminton offered modest opportunities that were more accessible and tangible, especially highlighted through the many athletes who started from marginalized regions. Participants reported more straightforward paths to success, obtaining tangible benefits such as stipends and institutional support alongside social benefits such as networking. Although not a panacea, badminton delivered unique values in marginalized communities by effectively opening incremental avenues for advancement where few such outlets exist.

Bourdieu's lens helps in the interpretation of these divergent trajectories. In soccer's competitive field, those already endowed with economic, social, or cultural capital are positioned to accumulate further advantages. This dynamic perpetuates social reproduction, as underprivileged players who already have fewer resources find it even harder to improve social mobility within the competitive soccer hierarchy. Even dominant agents like elite clubs and other gatekeepers are revealed to exert uneven symbolic power. This narrows outsider opportunities, reinforcing the status quo. In contrast, the emergent field of badminton provides a significantly more level playing ground. Participants are found to acquire sporting capital and convert modest achievements into educational or social gains with substantially fewer barriers. In Bourdieu's terms, the niche sport with lower participation rates allowed for fewer variations in athletes' initial habitus. Similarly, the higher accessibility allows for less *impact* of habitus; being located in a more “elite” region offers less benefits as it would in soccer. It has thus been observed that the niche field contained lowered internal inequalities and improved capital accumulation that would be elusive in the tightly regulated soccer field.

Sen's capability approach further highlights these patterns by complementing the field-capital lens. Participation did not broaden capabilities for most soccer players, especially the ones already experiencing marginalization. Many players experienced failed social relations, which led to capability deprivation as their engagement yielded few gains in valued functionings like education, employment, and wages. On the other hand, involvement in badminton coincided with smaller but tangible capability gains. The niche sport offered a broader set of achievable life alternatives, contrasting soccer's winner-takes-all model. This aligns with Sen's notion of development as freedom, illustrating the potential of niche sports in bolstering social mobility.

## 7 Limitations and future research

The comparative analysis is designed for analytical depth over breadth. However, it is important to explicitly note the limitations in the study. First of all, the primary dataset I used consists of three semi-structured interviews. This narrows the range of voices that are being investigated, potentially introducing selection effects. Furthermore, the contrast between one mainstream and one niche sport leads to constraints on external validity, so the findings should not be read as estimating prevalence or magnitudes across Latin America's diverse sport ecologies. They serve instead as indicators that niche sports hold value for future attention. These constraints also limit statistical generalization, which precludes population-level claims about outcomes. Nonetheless, as mentioned priorly, this study actively works to mitigate these risks in order to provide confidence and validity in the results. Interview testimonies are corroborated with policy documents, federation and government programs, media reports, as well as independent organizational accounts from 47 core sources. Cases are also treated as illustrative rather than representative. All interpretations are furthermore anchored in Bourdieu's field-capital and Sen's capability to conversion frameworks, supporting an analytic mechanism-level generalization even when statistical generalization is not possible. Future research may broaden the provided evidentiary base via multi-country and multi-sport sampling. This includes but is not limited to (i) compiling and exploring larger primary datasets to help capture non-elite and dropout trajectories (data here is currently limited); (ii) mixed-methods approaches like the inclusion of longitudinal panels to track educational attainment, employment quality, and mental health effect differences; (iii) quasi-experimental evaluations of thresholds for stipends alongside examination into program rollouts like Bolsa Atleta; and (iv) an egocentric network mapping for the quantification of social capital. This study ultimately acts as an initiating point for a broader line of Latin American niche-sport research, and I hope that it inspires subsequent investigation to strengthen and reveal further patterns in this novel approach to SFD.

## Data Availability

The original contributions presented in the study are included in the article/Supplementary Material, further inquiries can be directed to the corresponding author.

## Footnotes

1 Cordón K. Interview by O. Chen and G. Buendía, Guatemala City, July 11, 2025. Personal interview. (2025).
